# Economic assessment of starting robot-assisted laparoscopic inguinal hernia repair in a single-centre retrospective comparative study: the EASTER study

**DOI:** 10.1093/bjsopen/zraa046

**Published:** 2021-01-08

**Authors:** F Muysoms, M Vierstraete, F Nachtergaele, S Van Garsse, P Pletinckx, A Ramaswamy

**Affiliations:** Department of Surgery, Maria Middelares Hospital, Ghent, Belgium; Department of Surgery, Maria Middelares Hospital, Ghent, Belgium; Department of Surgery, Maria Middelares Hospital, Ghent, Belgium; Department of Medical Direction, Maria Middelares Hospital, Ghent, Belgium; Department of Surgery, Maria Middelares Hospital, Ghent, Belgium; Department of Surgery, University of Minnesota, Minneapolis VA Medical Center, Minneapolis, Minnesota, USA

## Abstract

**Background:**

There has been a rapid adoption of robot-assisted laparoscopic inguinal hernia repair in the USA, despite a lack of proven clinical advantage and higher material cost. No studies have been published regarding the cost and outcome of robotic inguinal hernia surgery in a European Union setting.

**Methods:**

A retrospective comparative study was performed on the early outcome and costs related to laparoscopic inguinal hernia repair, with either conventional or robot-assisted surgery.

**Results:**

The study analysed 676 patients undergoing laparoscopic inguinal hernia repair (272 conventional and 404 robotic repairs). Conventional laparoscopic and robotic repair groups were comparable in terms of duration of surgery (57.6 *versus* 56.2 min respectively; *P* = 0.224), intraoperative complication rate (1.1 *versus* 1.2 per cent; *P* = 0.990), in-hospital complication rate (4.4 *versus* 4.5 per cent; *P* = 0.230) and readmission rate (3.3 *versus* 1.2 per cent; *P* = 0.095). There was a significant difference in hospital stay in favour of the robotic approach (*P* = 0.014), with more patients treated on an outpatient basis in the robotic group (59.2 per cent *versus* 70.0 per cent for conventional repair). At 4-week follow-up, equal numbers of seromas or haematomas were recorded in the conventional laparoscopic and robotic groups (13.3 *versus* 15.7 per cent respectively; *P* = 0.431), but significantly more umbilical wound infections were seen in the conventional group (3.0 per cent *versus* 0 per cent in the robotic group; *P* = 0.001). Robotic inguinal hernia repair was significantly more expensive overall, with a mean cost of €2612 *versus* €1963 for the conventional laparoscopic approach (mean difference €649; *P* < 0.001).

**Conclusion:**

Robot-assisted laparoscopic inguinal hernia repair was significantly more expensive than conventional laparoscopy. More patients were treated as outpatients in the robotic group. Postoperative complications were infrequent and mild.

## Introduction

Robot-assisted laparoscopic inguinal hernia repair (IHR) has seen a rapid adoption in the USA despite lack of proven clinical advantage and higher material cost^1–3^. Outcomes of individual studies are not homogeneous regarding the clinical benefits of adopting robotic IHR, and the large majority of the studies have been retrospective, thereby decreasing the validity of their results.

The strongest data supporting robotic IHR come from a database study showing significantly fewer complications compared with open or laparoscopic IHR^4^. A systematic literature review^5^ from 2018 also noted a lower postoperative complication rate for robotic compared with open IHR, but found no difference between robotic and laparoscopic IHR. Another study^6^, comparing open and robotic IHR, showed a lower 30-day complication rate after robotic IHR. Data from the Americas Hernia Society Quality Collaborative^3^ demonstrated low and comparable rates of 30-day complications between open, laparoscopic and robotic IHR of unilateral uncomplicated inguinal hernias. In addition, several other studies^2^^,^^7–10^ have found no difference in early clinical outcome for laparoscopic *versus* robotic IHR. Conversely, one smaller, single-centre study^11^ noted worse clinical outcomes after robotic IHR, with an increased rate of severe complications compared with laparoscopic IHR. Only one published randomized study^12^ has compared robotic and laparoscopic IHR; it found no significant clinical benefit at 30 days after surgery.

In contrast with clinical outcomes, there is much less heterogeneity regarding the cost related to adopting robotic IHR compared with laparoscopic IHR. The overall cost of robotic IHR is higher, but with a wide variation in excess between studies^2^^,^^7^^,^^11–13^, ranging from $926 to $3999 (€774 to €3342, exchange rate 1 December 2020) . This overall cost is highly dependent on the methodology used to calculate the costs, with the excess cost attributable mainly to a higher material cost and longer operating time. In most studies, the capital cost of the robotic platform is not included in the comparison. Longer operating times were reported for robotic IHR compared with laparoscopic IHR in most studies^2^^,^^7^^,^^11–13^, but the effect of the learning curve must be considered when using duration of surgery as a variable in cost calculations. Studies^8^^,^^9^^,^^14^ have demonstrated similar operating times between robotic and laparoscopic IHR after the learning curve.

No published studies have investigated the cost and outcome of robotic IHR in a European Union setting. The aim of the present study was to determine the additional hospital cost incurred when performing laparoscopic groin hernia repair with the da Vinci^®^ Xi system (Intuitive, Sunnyvale, CA, USA), compared with a technique using conventional laparoscopic instruments.

## Methods

This study was undertaken in accordance with the STROBE statement^15^.

### Design, setting and participants

This was a retrospective single-centre comparative study of endoscopic IHR using conventional laparoscopy or robot-assisted laparoscopy. The study was performed at the Department of Surgery of Maria Middelares Hospital in Ghent, Belgium. All operations were performed by one surgeon with extensive experience in laparoscopic IHR. The robotic IHR programme commenced in September 2016. The study population comprised all patients operated on from January 2016 to December 2019. The study protocol was submitted to ClinicalTrials.gov (NCT04431271) before the start of the study (June 2020).

In Belgium, IHR is reimbursed by governmental medical insurance, which is a single-payer system. In this system, hospital stay, physician fees, mesh cost and consumables are reimbursed separately. The reimbursed cost for hospital stay is a fixed amount of €595 per day for the hospital. When patients are treated on an outpatient basis, the hospital receives only 80 per cent of this amount. This fixed amount covers nursing, hospital infrastructure, hospital staff and generic consumables (needles, sutures, operating room equipment). Physician fees are transferred to the surgeon once hospital overheads have been deducted, as a percentage. For consumables, a fixed fee, depending on the surgical procedure performed, is reimbursed. For IHR, three fixed fees are paid. A first fee covers the pharmacy costs, excluding the mesh or surgical instruments, at a flat daily rate. The second covers synthetic mesh used during surgery. A third flat rate reimbursement covers the material used during the procedure, and is procedure-specific. For laparoscopic IHR, the flat rate is €716, regardless of whether the procedure is completed via a conventional or robot-assisted approach. The fee also remains unchanged if unilateral or bilateral hernias are repaired.

Adult patients who underwent conventional or robot-assisted laparoscopic IHR were included in the study. The following were excluded from the data set: patients who had an open inguinal hernia repair and those who underwent a concurrent additional procedure.

All patients were scheduled for a standard clinical outpatient follow-up visit with the surgeon at 4 weeks after surgery.

### Surgical technique

All operations were performed under general anaesthesia. No prophylactic antibiotics were given. Urinary catheters were placed in patients with a history of abdominal prostatectomy. Patients were instructed to void before surgery. Hernia repair was performed according to standard surgical principles, with mesh placement after appropriate preperitoneal dissection once the critical view of the myopectineal orifice had been achieved^16^^,^^17^.

#### Conventional laparoscopic inguinal hernia repair

A laparoscopic 0° camera with three-dimensional visualization (Karl Storz, Tuttlingen, Germany) was used with an 11-mm disposable trocar at the umbilicus and two 5-mm disposable trocars for instruments on both sides of the umbilicus (trocars from Ethicon, Johnson & Johnson, Somerville, NJ, USA). Pneumoperitoneum was created with a Veress needle, with pressure maintained at 12 mmHg. Self-gripping monofilament polyester mesh (Parietex Progrip™ Self-Fixating Mesh; Medtronic, Minneapolis, MN, USA) was used, with a width of 16 cm and length of 12 cm for unilateral hernias, and a width of 28 cm and length of 13 cm for bilateral hernias. Care was taken to close the peritoneum properly after mesh placement using a barbed suture (V-Loc™ 90; Medtronic). The fascia at the umbilical trocar site was closed with a U stitch of absorbable suture, size 0 (Vicryl^®^ Plus; Ethicon, Johnson & Johnson). Two semireusable endograspers were used, in addition to disposable endoscopic scissors with monopolar cautery, and a reusable needle-driver.

#### Robot-assisted laparoscopic inguinal hernia repair

Robotic operations were performed using the da Vinci^®^ Xi system with a 0° scope. Trocar positions were identical to those for laparoscopic IHR, but the trocars were all 8 mm in size. Blind entry of the blunt first trocar at the umbilicus was performed to create the pneumoperitoneum. Surgical technique, including mesh choice and peritoneal flap closure, was identical to that used in the conventional laparoscopic approach. During the start-up period of the robotic programme, two separate meshes were used for bilateral IHR. Following increased experience with the robotic platform, one mesh was used to cover both groins. The 8-mm trocar fascial defects were not closed. Two robotic instruments were used for the majority of operations. Additional instruments were used for more complex situations. These instruments included hot shears monopolar curved scissors, fenestrated bipolar forceps, and a large needle-driver.

### Outcomes

The primary endpoint of the study was the overall cost of endoscopic IHR, including costs for hospital stay, physician fees and consumables. Secondary endpoints were the duration of surgery (timed in minutes from first skin incision to last skin stitch), intraoperative complications, postoperative complications during the hospital stay (graded according to the Clavien–Dindo classification^18^), urinary retention, hospital stay (stratified as surgery as an outpatient, 1-night stay, stay longer than 1 night), number of readmissions related to the IHR within 6 weeks of the operation date, and complications recorded at the 4-week follow-up visit (grouped as seroma–haematoma, wound infection, or other). All patient and surgical variables were entered prospectively into the European Registry of Abdominal Wall Hernias (EuraHS) online database^19^ at the time of surgery and at the 4-week follow-up. The European Hernia Society (EHS) classification of groin hernias^20^ was used.

The cost of hospital stay was provided by the hospital accounting department. Physician fees were extracted from billing data. Consumable costs were extracted from the pharmacy record. This included the costs of pharmaceuticals, the mesh, and the endoscopic material used during surgery. This material includes disposable items (Veress needle, disposable laparoscopic trocars, seals for reusable robotic trocars, plastic drapes for robotic arms) and reusable items (laparoscopic graspers and needle-holder and robotic instruments that have 10 uses). Cost analysis did not include the capital cost of the laparoscopic or robotic systems used. All costs are described in euros (€).

### Statistical analysis

Data analyses were performed by an independent statistician. The distributions of patient characteristics, operative data and postoperative complications were summarized using proportions, mean or median. Differences between groups were evaluated statistically with Fisher's exact test or the Mann–Whitney *U* test. A multivariable model was established associating the total costs (in €) with the IHR approach (robotic *versus* conventional laparoscopy), adjusting for perisurgical characteristics. Costs in this model were ln-transformed to meet linear model assumptions. The model was free from multicollinearity, and model assumptions were checked by visual inspection of the Pearson residuals. A type I error level of α = 0.05 was chosen to indicate statistical significance. All analyses were performed using SAS^®^ software release 9.4 (SAS Institute, Cary, NC, USA).

## Results

**Fig. 1:**
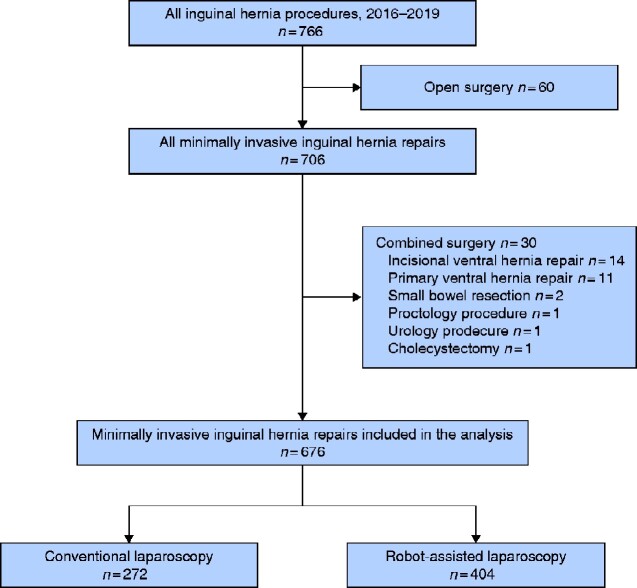
Flow diagram showing inclusion of patients in the study

Between January 2016 and December 2019, 766 patients underwent IHR. After excluding open repairs (*n* = 60) and combined operations (*n* = 30), a total of 676 patients were included in the analysis: 272 laparoscopic and 404 robotic IHRs (*Fig. 1*).

Patient and hernia characteristics at baseline are shown in *Table 1*. The groups are comparable for all variables except for sex and hernia size. More women underwent conventional laparoscopic IHR. Of note, the mean cost of IHR did not differ between women and men (€2333 *versus* €2352; *P* = 0.262). More patients in the robotic IHR group had EHS hernia size 2 and fewer had EHS hernia size 1. No difference in EHS hernia size 3 was detected between the groups.

**Table 1:** Baseline data and surgical characteristics

	Conventional IHR (*n* = 272)	Robotic IHR (*n* = 404)	***P*** ^‡^
**Age (years)** *	60.3 (62.0)	60.0 (61.7)	0.651^§^
**No. of women**	35 (12.9)	27 (6.7)	0.009
**Hernia side**			0.883
Bilateral	127 (46.7)	190 (47.0)	
Left	68 (25.0)	106 (26.2)	
Right	77 (28.3)	108 (26.7)	
**Recurrent hernia**	17 (6.3)	32 (7.9)	0.452
**History of abdominal prostatectomy**	1 (0.4)	13 (3.2)	0.011
**Emergency surgery**	6 (2.2)	8 (2.0)	0.890
**Hernia grade** ^†^			0.036
1	56 (20.7)	57 (14.1)	
2	161 (59.4)	276 (68.3)	
3	54 (19.9)	71 (17.6)	
Missing	1	0	
**Hernia location**			
Medial	114 (41.9)	188 (46.5)	0.236
Lateral	192 (70.6)	285 (70.5)	0.990
Femoral	20 (7.4)	27 (6.7)	0.737
**Anticoagulation**	48 (17.6)	70 (17.3)	0.918
**Smoker**	45 of 271 (16.6)	56 of 403 (13.9)	0.379
**History of previous hernia surgery**	45 (16.5)	79 (19.6)	0.362
**COPD**	21 (7.7)	28 (6.9)	0.763
**Diabetes mellitus**	11 (4.0)	25 (6.2)	0.295
**Renal kidney disease**	5 (1.8)	6 (1.5)	0.763
**Abdominal aortic aneurysm**	2 (0.7)	2 (0.5)	0.990
**Hepatic liver disease**	1 (0.4)	2 (0.5)	0.990
**BMI (kg/m^2^)**			0.552
<25	142 (52.2)	205 (50.9)	
25–30	109 (40.1)	174 (43.2)	
>30	21 (7.7)	24 (6.0)	
Missing	0	1	

Values in parentheses are percentages unless indicated otherwise:

*values are mean (median).

^†^European Hernia Society classification^20^: grade 1, below 1.5 cm; grade 2, 1.5–3 cm; grade 3, more than 3 cm. IHR, inguinal hernia repair; COPD, chronic obstructive pulmonary disease.

^‡^Fisher's exact test, except

^§^Mann–Whitney *U* test.

Clinical outcomes are shown in *Table 2*. No difference in duration of surgery, intraoperative complications, postoperative in-hospital complications, or postoperative urinary retention was found. Hospital stay was significantly shorter in the robotic IHR group with a higher proportion of patients treated on an outpatient basis. More patients were readmitted after discharge in the laparoscopic group, but the difference was not significant (*P* = 0.095). At 4-week follow-up, a higher rate of superficial wound infections at the umbilicus was seen in the conventional laparoscopic IHR group (*P* = 0.001). The majority of these eight patients were treated on an outpatient basis by their primary care physician, with removal of skin sutures with or without oral antibiotics. One patient required readmission for intravenous antibiotics.

**Table 2:** Clinical outcome data

	Conventional IHR (*n* = 272)	Robotic IHR (*n* = 404)	***P*** ^‡^
**Duration of surgery (min)** ^*^			
Overall	57.6 (54.0)	56.2 (53.0)	0.224^§^
Unilateral hernia	50.0 (47.0)	48.4 (44.5)	0.170^§^
Bilateral hernia	66.2 (62.0)	65.0 (60.0)	0.519^§^
**Intraoperative complications**	3 (1.1)	5 (1.2)	0.990
**In-hospital complications** ^†^			0.230
No complications	260 (95.6)	386 (95.5)	
Grade I	1 (0.4)	3 (0.7)	
Grade II	7 (2.6)	14 (3.5)	
Grade IIIa	3 (1.1)	0 (0)	
Grade IIIb	1 (0.4)	1 (0.2)	
Grade IV	0 (0)	0 (0)	
Grade V	0 (0)	0 (0)	
**Postoperative urinary retention**	10 (3.7)	14 (3.5)	P0.990
**Hospital stay**			0.014
Outpatient treatment	161 (59.2)	283 (70.0)	
1 night	95 (34.9)	102 (25.2)	
≥2 nights	16 (5.9)	19 (4.7)	
**Readmission**	9 (3.3)	5 (1.2)	0.095
**Follow-up at 4 weeks**			
Lost to follow-up	9 (3.3)	16 (4.0)	0.834
4-week follow-up	*n* = 263	*n* = 388	
No complications	215 (81.7)	323 (83.2)	0.673
Seroma or haematoma	35 (13.3)	61 (15.7)	0.431
Wound infection	8 (3.0)	0 (0.0)	0.001
Other	5 (1.9)	4 (1.0)	0.496

Values in parentheses are percentages unless indicated otherwise:

*values are mean (median).

^†^Grading according to Clavien–Dindo classification^18^. IHR, inguinal hernia repair.

^‡^Fisher's exact test, except

^§^Mann–Whitney *U* test.

**Fig. 2:**
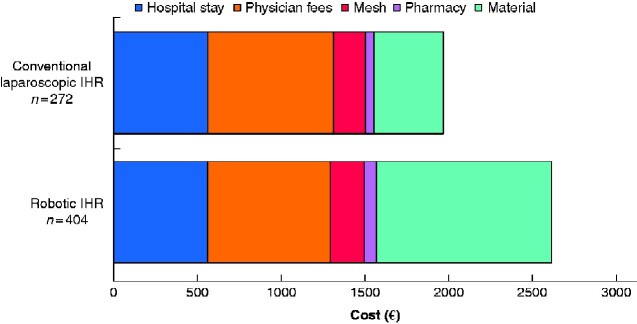
Bar chart depicting cost data for conventional and robot-assisted laparoscopic inguinal hernia repair IHR, inguinal hernia repair.

*Table 3* shows the cost data. Robotic IHR was more expensive than conventional laparoscopic IHR, with a mean cost difference of €649 (*P* < 0.001). This higher cost was related mainly to the higher cost of materials used during surgery. In addition, the mean cost of mesh was slightly higher for robotic IHR (mean cost difference €14; *P* < 0.001). The results are depicted graphically in *Fig. 2*. There were 14 readmissions, with a mean cost per patient for readmission of €3441. When the costs for procedure-related readmissions were included in the calculation, the mean cost difference between robotic and conventional laparoscopic IHR decreased to €575 (*Table 3*).

**Table 3:** Cost data for the index operation and index operation plus readmissions

	Conventional IHR (*n* = 272)	Robotic IHR (*n* = 404)	Δ robotic *versus* conventional IHR
**Index operation costs (€)**			
Hospital stay	564	561	−4
Physician fees	751	734	−17
Consumables	648	1317	+669
Mesh	187	201	+14
Pharmacy	54	77	+23
Material	408	1039	+632
Overall	1963	2612	+649
**Index operation + readmission costs (€)**			
Hospital stay	635	590	−45
Physician fees	784	742	−42
Consumables	659	1321	+662
Mesh	187	201	+14
Pharmacy	59	79	+20
Material	413	1041	+628
Overall	2078	2653	+575

Values show the mean cost per patient. IHR, inguinal hernia repair.

A multivariable analysis associating perisurgical characteristics with total cost was performed. The total cost was increased significantly with emergency surgery, bilateral groin hernia repair, robot-assisted surgery, postoperative complications of grade III or above, and increased hospital stay. *Table 4* shows the cost of material used for conventional *versus* robot-assisted IHR and the reimbursement received by the hospital from government medical insurance, specifically for the cost of material during laparoscopic IHR. Two robotic instruments were used for the majority of operations (78.7 per cent). Additional instruments were used for more complex situations (20.8 per cent).

**Table 4:** Costs of material used during laparoscopic inguinal hernia repair with a conventional or robot-assisted approach

	**Cost (€)** ^*^	**Costing 1 (€)**	**Costing 2 (€)**
**Conventional IHR**			
Instrument or item		No fixation	Stapler fixation
Veress needle	18.3	1	1
Trocar kit (11 mm and 2×5 mm)	203.2	1	1
Set of 2 reusable graspers	115.1	1	1
Laparoscopic scissors	47.8	1	1
Reusable needle-driver	–	1	1
Glue	290.0		
Stapler	376.2		1
Total instrument cost		384.4	760.6
Δ with government reimbursement at €716		+331.6	−44.6
**Robotic IHR**			
Instrument or item		2 instruments	3 instruments
Hot shears curved scissors	387.2	1	1
Tip cover	24.2	1	1
Large needle-driver	266.2	1	1
Fenestrated bipolar forceps	326.7		1
ProGrasp™ forceps	266.2		
Arm drape	62.9	3	3
Column drape	21.8		
Universal seal (5–8 mm)	18.1	3	3
Reusable grasper	24.2	1	1
Total instrument cost		944.8	1271.5
Δ with government reimbursement at €716		−228.8	−555.5

*Mean cost per patient. Da Vinci^®^ ProGrasp™ forceps (Intuitive, Sunnyvale, CA, USA). IHR, inguinal hernia repair.

This retrospective comparative study analysed 676 patients undergoing laparoscopic inguinal hernia repair: 272 conventional and 404 robotic repairs. Robotic repair was significantly more expensive than conventional laparoscopy, but allowed more patients to be operated on an outpatient basis. Postoperative complications were infrequent and mild, with a slightly higher readmission rate for laparoscopic repairs.

**Fig. 3:**
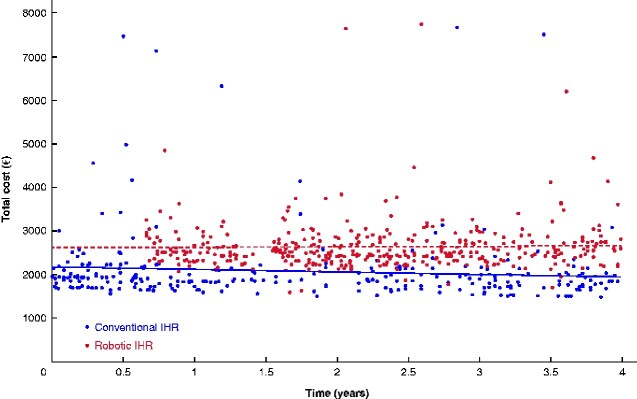
Cost data over time (January 2016 to December 2019) for conventional and robot-assisted laparoscopic inguinal hernia repair IHR, inguinal hernia repair.

*Fig. 3* shows the evolution over time of the total cost for IHR performed with conventional laparoscopy or robot-assisted surgery, and illustrates that the difference in cost was maintained and stable over time.

## Discussion

Robotic IHR had a higher cost of €649 compared with conventional laparoscopic IHR. Operating times were similar and the early clinical outcome was excellent for both approaches, with a greater proportion of patients being treated on an outpatient basis for the robotic approach.

The excess cost of €649 calculated for the authors’ hospital was lower than has been found in most studies from the USA, where cost differences have ranged from $926 to $3999 (€774 to €3342)^2^^,^^7^^,^^11–13^. European healthcare systems and financing are very different from the US models. There is also a significant degree of heterogeneity between healthcare systems across Europe. Some countries have a fixed flat rate for inguinal hernia repair regardless of technique. Some systems financially encourage treatment on an outpatient basis, whereas others do not. The best financial model for a hospital in certain healthcare systems is inpatient IHR. Belgium has a single-payer model, in which hospitalization, physician fees, mesh cost and materials are reimbursed separately. Physician fees are not different between robotic and conventional laparoscopic IHR. The present study did find a higher mean cost for mesh in the robotic IHR group, with a mean cost difference of €14. This was related to the use of two separate meshes for bilateral IHR in the initial period of robotic platform use, as this appeared to be technically easier. The cost for a unilateral mesh of 16×12 cm is €194, and thus bilateral repairs using two separate meshes incurred a cost of €388 per patient. After achieving more proficiency with the robotic platform, the technique was changed to the use of one large mesh of 28×13 cm for bilateral hernias, as in conventional laparoscopic IHR. This was a cheaper option, with the cost for one large mesh being €198 per patient.

Cost analysis did not include the capital cost of the laparoscopic or robotic systems used. It is difficult to amortize the cost of equipment for different procedures.

Material costs were higher for robotic IHR, and this difference was not offset completely by the reduction in hospital stay in the authors’ system. Similarly, the statistically non-significant higher rate of readmission for conventional laparoscopic IHR did not recoup the cost difference.

Belgium has a specific reimbursement of €716 for the material cost of IHR performed laparoscopically. Although this fixed rate is higher than the actual material cost of conventional laparoscopic IHR (€408) in the authors’ hospital, it is lower than the cost of robotic IHR (€1039). In the authors’ specific situation, 50 per cent of IHRs would need to be performed via conventional laparoscopy and 50 per cent robotically for the hospital to avoid incurring a net loss. Operating time was not included in the cost calculations, as there was no difference in duration of surgery between the groups. In the only RCT published on the topic, Prabhu and colleagues^12^ used operating time to calculate the operating room cost, and this amounted to almost one-third of their excess cost for robotic IHR. It is important to note that the techniques differed between the arms in their study^12^, with staple fixation for conventional IHR *versus* a more time-consuming suture fixation for robotic IHR. The present authors^8^ and others^9^ have demonstrated that duration of surgery is not longer with the robotic approach after the learning curve.

A reduction in complications could be a reason to justify a higher procedure cost; however, no difference in the early complication rate was found for robotic IHR in the present study. There was an increase in the number of superficial wound infections at the umbilicus for conventional laparoscopic IHR. The umbilical trocar used for conventional laparoscopic IHR was 11 mm in size and the fascia was closed with a suture, whereas the fascia of the 8-mm trocars used for robotic IHR was not closed. The authors’ practice is to close trocar defects of 10 mm or more with a fascial stitch of a multifilament absorbable suture (Vicryl^®^ Plus). Use of a monofilament suture might decrease the risk of wound infection, and is being considered. Manipulation around the umbilical trocar is decreased with the robotic approach after docking. It is not clear whether the above points account for the difference noted. Nevertheless, all wound infections at the umbilical incision were mild, and only one patient required readmission for intravenous antibiotics.

The cost of the robotic instruments appears to be too high to allow generalized adoption of a robotic platform for the treatment of all groin hernias in a European Union setting. The authors believe that robotic IHR may have benefits for patients with a complex inguinal hernia, owing to the advantages of the robotic platform: better visualization, availability of wristed instruments, and a more stable operating field, as described. Moreover, they consider robotic IHR an important index procedure in the training of surgeons using the robotic platform to repair abdominal wall hernias. The benefits of the robotic system are probably more notable for patients undergoing more complex ventral hernia repair (retrorectus and component separation techniques). However, gaining the skill set to perform these procedures is important, and the less technically challenging robotic IHR introduces the techniques of dissection, suturing and mesh handling on the abdominal wall.

Some modest changes would significantly alter the value proposition for robotic IHR. The present data suggest that a cost reduction for currently available robotic instruments of 30 per cent, or increased use of the individual robotic instruments to 15 (as opposed to the current 10) uses would allow the costs of robotic IHR to match the reimbursement.

This was a retrospective study with the known inherent limitations of this design. The study included some historical data for conventional IHR before the start of robotic IHR in September 2016. With increased experience with the robotic platform, indications for the laparoscopic approach were extended to include complex IHR after prostatectomy, large inguinoscrotal inguinal hernias, and recurrences after previous preperitoneal mesh repair. The proportion of open IHR cases decreased from 17 to 6 per cent between 2015 and 2019. Selection bias was most likely present, as patients with more complex hernias were consciously chosen for robotic repair. Other than the scenarios mentioned above, the selection for robotic or conventional laparoscopic IHR was determined by access to the robotic platform on the day of the planned surgery. Misclassification bias may also have been present, specifically with documentation of instrument use in the operating room. The authors’ hospital is very cost-conscious, and the authors are very conservative with the utilization of consumables in the operating room. This cost-containment culture might differ from that in other institutions. Although all these procedures were performed or supervised by the same surgeon, residents and fellows may have performed the procedures partially or completely.

These results are applicable to the authors’ centre, a medium-sized Belgian hospital that focuses on cost containment, but within an environment that strongly supports innovation. The economic equation of cost and hospital income will differ significantly between countries inside the European Union. These cost calculations were specific to a single robotic platform, and will be different for robotic systems that will enter the market in the near future.

## Funding

Medical writing support agreement for Research

Intuitive
